# PI-3 kinase activity is necessary for ERK1/2-induced disruption of mammary epithelial architecture

**DOI:** 10.1186/bcr2259

**Published:** 2009-05-20

**Authors:** Gray W Pearson, Tony Hunter

**Affiliations:** 1 Molecular and Cell Biology Laboratory, Salk Institute, 10010 N. Torrey Pines Road, La Jolla, CA 92037, USA

## Abstract

**Introduction:**

Epithelial tumors, including breast cancer, are being identified and treated at earlier stages of tumor development because of technological advances in screening and detection methods. It is likely that early-stage epithelial tumors, such as mammary ductal carcinoma *in situ *(DCIS), will be amenable to new and more efficacious diagnostic tests and forms of therapy. However, our limited understanding of the underlying molecular mechanisms of early-stage epithelial tumor growth has hampered the development of new forms treatment and preventative therapy.

**Methods:**

The Raf–MEK1/2–ERK1/2 mitogen-activated protein kinase module is activated by stimuli complicit in mammary neoplastic progression. We have recently demonstrated that the activation of ERK1/2 induces a non-invasive form of motility, where cells can track along the basement membrane and adjacent epithelial cells, but do not become invasive over time, using real-time imaging of a mammary epithelial organotypic culture model. Using this novel approach combined with traditional biochemical techniques, we have analyzed at the molecular level how ERK1/2 induces this new non-invasive form of motility as well as proliferation and cell survival.

**Results:**

We find that the activation of Raf:ER in the differentiated epithelium of fully formed acini promotes proliferation and cell survival, which are characteristic features of pre-invasive DCIS lesions. The activation of ERK1/2 correlated with induction of c-Fos, a transcriptional regulator of proliferation and reduced expression of the pro-apoptotic BH3-only protein BIM. Both ERK1/2 and PI-3 kinase-dependent effector pathways were required for activated Raf:ER to reduce expression of p27 and promote proliferation. In addition, PI-3K activity was necessary for the induction of non-invasive motility induced by ERK1/2.

**Conclusions:**

ERK1/2 activation is sufficient to induce cell behaviors in organotypic culture that could promote recurrent and invasive growth in DCIS patients. Interestingly, PI-3K activity is necessary for two of these behaviors, proliferation and cell motility. Collectively, our results suggest that the relationship between the activity state of the ERK1/2 and PI-3K signaling pathways and recurrent growth in DCIS patients should be investigated.

## Introduction

Epithelial cancers, such as breast cancer, are being more frequently identified at the early pre-invasive stage of tumor development [1]. These pre-invasive mammary lesions originate from the luminal epithelial cells that line the ducts and lobules of the mammary glandular epithelium and have a disrupted epithelial architecture characterized by hyperproliferative cells occupying the normally hollow luminal spaces of the ducts and lobules [2,3]. The amplification and overexpression of the receptor tyrosine kinase ErbB2 is observed in approximately 50% of pre-invasive lesions; however, in most cases, the genetic and epigenetic abnormalities that promote pre-invasive tumor growth are poorly understood [4].

Since such a wide range of molecular perturbations can induce and enhance tumor growth, there are probably shared molecular signaling modules that integrate biochemical signals from the suite of genetic contexts found in epithelial tumors [5]. To explain how normal cells become tumorigenic, a molecular framework that underpins the pre-invasive stage of tumor growth must be established. Such a molecular framework can assist in the identification of patients amenable to targeted therapeutics, in the development of novel therapeutics to treat pre-invasive cancer, and, in the future, in the introduction of preventative treatment [6]. Attempts to identify the core signaling modules that promote these pre-invasive growth characteristics through the analysis of genetic abnormalities and gene expression patterns of pre-invasive tumor lesions have to date been unsuccessful [7-9].

The Raf–MEK1/2–ERK1/2 mitogen-activated protein kinase signal transduction module transmits extracellular and oncogenic stimuli, resulting in cellular responses [10]. In this module, Raf isoforms phosphorylate their primary substrates, the dual-specificity kinases MEK1/2. Once activated, MEK1/2 phosphorylate ERK1/2 on tyrosine and threonine residues, substantially increasing ERK1/2 catalytic activity [11]. The Raf–MEK1/2–ERK1/2 module is activated by growth factors and proteins overexpressed in human breast cancer epithelium, by cytokines and hormones produced by fibroblasts and macrophages in the mammary stromal compartment, and by increased tissue stiffness observed during tumor progression [10,12]. In addition, the sequencing of breast cancer patient genomes suggests that infrequent mutations may drive tumor progression through known signaling pathways, such as the Raf–MEK1/2–ERK1/2 cascade [5]. Considering the array of stimuli known to activate the Raf–MEK1/2–ERK1/2 module, it may be complicit in tumorigenesis in a variety of contexts.

Consistent with a role for the Raf–MEK1/2–ERK1/2 module in mammary carcinogenesis, ERK1/2 are activated in primary breast cancer tissue and in associated lymph node metastases [13,14]. The activation of ERK1/2 is not associated with a specific genetic signature, however, as ERK1/2 is active in ER-positive breast cancer, HER2-positive breast cancer and in triple-negative breast cancer [15]. ERK1/2 phosphorylate transcription factors, kinases, proteases and non-enzymatic regulatory proteins, thus potentially integrating the Raf–MEK1/2–ERK1/2 module into a range of cellular activities associated with tumorigenesis [16]. Accumulating evidence, however, has shown that results obtained in one cell type should not be generally applied across all classes of cancer without experimental validation [6]. For example, the K-Ras2 oncogene has distinct effects on tumor progression depending on both the cell type of origin and the genetic context in which it is mutated [6]. In addition, extrapolating the role of protein kinases in promoting breast cancer progression based on either their known substrate profile or biological behaviors induced in two-dimensional culture models has proven to be unreliable [17,18]. For example, the chemically induced homodimerization of the epidermal growth factor receptor (EGFR) is sufficient to induce focus formation in Rat 1 cells and the proliferation of MCF-10A mammary epithelial cells in monolayer cultures [17,19]. EGFR homodimerization of EGFR, however, is not sufficient to induce the proliferation of differentiated MCF-10A cells grown in organotypic culture [17]. Considering the uncertainty in predicting the response of cells to the activation of a signaling pathway, determining the response of differentiated mammary epithelial cells to Raf–MEK–ERK activation can better define the early events of mammary tumorigenesis.

Three-dimensional organotypic culture models have been indispensable tools in deciphering the molecular and cell biological mechanisms underlying the disruption of differentiated epithelial architecture that is characteristic of pre-invasive mammary epithelial lesions. In organotypic culture models, individual mammary epithelial cells plated on reconstituted basement membrane proliferate to form a hollow sphere of polarized, growth-arrested cells (termed acini), thus recapitulating the salient features of the mammary gland [20,21]. Since the mammary epithelial cells differentiate and form a hollow monolayer of cells, organotypic cultures provide a more accurate reconstitution of the biochemical and cell biological growth restraints found in mammary glandular epithelium than is achieved using traditional two-dimensional cell culture models [22]. Once cells become proliferative, they are confronted with similar local environmental selection pressures to those found during tumorigenesis. Namely, cells are required to become resistant to cell death triggered by the induction of either apoptosis or autophagy when cells enter the luminal space [23,24]. Organotypic culture models therefore provide both the biochemical signaling barriers that must be overcome for initial proliferation to occur, and the microenvironmental context in which pre-invasive tumor cells must survive and propagate.

We have previously developed a method for imaging cells in Raf:ER-induced acini at single-cell resolution through imaging a histone–green fluorescence protein (GFP) correct fusion protein, H2B-GFP [25]. Using this unbiased discovery approach we have found that Raf:ER activation induces a disruption of epithelial architecture through promoting a non-invasive form of motility, cell proliferation and the survival of cells in the lumen. These findings suggest that ERK1/2 activation can promote the early events of tumorigenesis and that the induction of motility can, in principle, occur before tumor cell invasion. To determine how ERK1/2 signaling promotes the early events of tumorigenesis we have examined the intracellular signaling pathways that promote proliferation, cell survival and motility in response to ERK1/2 activation in mammary epithelial acini.

## Materials and methods

### Cell culture and reagents

MCF-10A human mammary epithelial cells were obtained from the American Type Tissue Culture Collection. Cells were cultured in DMEM/F12 (Gibco, Invitrogen, Carlsbad, CA, USA) supplemented with 5% horse serum (Gibco, Carlsbad, CA, USA), 10 μg/ml insulin (Research Diagnostics, Inc., Concord, MA, USA), 20 ng/ml epidermal growth factor (Research Diagnostics, Inc.), 500 ng/ml hydrocortisone (Sigma, St Louis, MO, USA), 100 ng/ml cholera toxin (Calbiochem, San Diego, CA, USA) and cyprofloxacin (Cellgro, Carlsbad, CA, USA). The growth-factor-reduced Matrigel (BD Biosciences, Franklin Lakes, NJ, USA) used in these experiments had protein concentrations between 10 and 12 mg/ml. 4-Hydroxytamoxifen (4-HT), LY294002, U0126 and AG1478 were from Calbiochem. Antibodies recognizing Ki-67 (Zymed, San Francisco, CA, USA), c-Fos, estrogen receptor alpha and cyclin B_1 _(Santa Cruz, Santa Cruz, CA, USA), phosphorylated AKT (S473), cleaved caspase 3, Bim and Bim (IF specific) (Cell Signaling, Beverly, MA, USA), p27 (BD Transduction Labs) and phosphorylated ERK2 (T183, Y185) (Sigma) were used. Secondary antibodies for immunofluorescence staining were labeled with Alexa fluor 488, 568 and 647 (Molecular Probes, Invitrogen, Carlsbad, CA, USA).

### Three-dimensional morphogenesis assay and cell lines

MCF-10A cells plated in eight-well chamberslides (Falcon, San Jose, CA, USA) were cultured as described previously [25]. The vector pBABE-Raf:ER was a gift from Michael White and Ron Bumeister (University of Texas Southwestern Medical Center, Dallas, USA), pBABE-GFP-Raf:ER was a gift from Martin McMahon (University of California San Francisco, USA) and pCLNRX-H2B:GFP was a gift from Ee Tsin Wong and Geoff Wahl (Salk Institute, La Jolla, CA, USA). VSVG-pseudotyped virus was generated by transfecting HEK293 cells stably expressing Gag and Pol with VSVG and pBABE-Raf:ER or pCLNRX-H2B:GFP. Cells were cultured in 500 ng/ml puromycin or 400 μg/ml G418 to create stable pools of pBABE-Raf:ER MCF-10A cells or pCLNRX-H2B:GFP MCF-10A cells. The GFP-Raf:ER MCF-10A cells did not undergo drug selection.

### Immunoblot analysis and immunofluorescence staining

The acini were lysed in RIPA buffer supplemented with protease and phosphatase inhibitors as described elsewhere [25], and protein levels were normalized using Cyto-tox One (Promega, Madison, WI, USA) according to the manufacturer's instructions. Immunoblots were visualized using an Odyssey infrared scanner (LI-COR, Lincoln, NE, USA). Cultures were fixed in 2% formalin (Sigma Aldrich, St. Louis, MO, USA) for 20 minutes and were permeabilized with 0.5% Triton X-100 in PBS for 10 minutes at room temperature. Immunostaining was performed as described previously [25].

Images were acquired on a Leica SP2 AOBS confocal microscope (Bannockburn, IL, USA) using Leica software in TIFF format. Images were arranged using Adobe Photoshop 7.0 and Keynote, and are representative of at least three independent experiments. For quantification of immunofluorescence images, either three or more Ki-67-positive cells per acinus or two or more phospho-AKT-positive cells per acinus were used as thresholds, as has been previously reported [17,24,26]. These thresholds reproducibly distinguish between control acini with normal architectures and Raf:ER-induced acini with disrupted architectures from experiment to experiment.

### Real-time imaging

Organotypic cultures were grown in eight-well chambered coverglass slides (Thermo Fisher Scientific, Pittsburgh, PA, USA) as described above and previously [25]. Cultures were imaged with a spinning disk confocal scanhead (QLC100; Yokagowa, Newnan, Georgia, USA) enclosed in a 37°C chamber supplemented with humidified carbon dioxide (Solent, Segensworth, UK) and a CCD camera (C9100-02 EM-charged coupled device; Hamamatsu, Hammamatsu, Japan). Images were acquired with a 40×/0.60 objective (HCX Plan Fluor; Leica) using SimplePCI software (Compix, Hammamatsu, Japan) and were analyzed with Imaris software (Bitplane, Zurich, Switzerland). At least six different *x*,*y *coordinates with three or more z-slices over 20 μm for each condition were imaged in parallel for three independent experiments.

## Results

### Activation of the Raf–MEK1/2–ERK1/2 mitogen-activated protein kinase module promotes increased proliferation and resistance to apoptosis

To elucidate how the Raf–MEK1/2–ERK1/2 module could promote pre-invasive tumor growth, we examined the response of a model human mammary epithelial cell line, MCF-10A, to activation of Raf in an organotypic culture model [25]. To activate Raf, a 4-HT-inducible, constitutively active variant of Raf-1, termed Raf:ER, was stably expressed in the MCF-10A cells [25,27]. The Raf:ER fusion protein consists of the kinase domain of Raf fused to a modified ligand-binding domain of the estrogen receptor at the C-terminus [28]. Treatment of cells with 4-HT activates Raf:ER by increasing Raf:ER protein stability and perhaps inducing conformational changes [29].

Using real-time imaging we have previously demonstrated that the activation of Raf:ER promotes the disruption of epithelial architecture of MCF-10A acini through the induction of a new non-invasive form of mammary epithelial cell motility [25]. In addition to cell motility, our real-time imaging analysis of Raf:ER-induced acini showed some cells transitioning through mitosis and that cells occupying the luminal space did not undergo apoptosis [25]. If Raf:ER induction was indeed inducing significant proliferation and cell survival, the size of acini should increase over time. To test this possibility we first grew Raf:ER-MCF-10A cells for 12 days in three-dimensional organotypic culture to generate acini with differentiated epithelium and a hollow lumen that are identical to wild-type MCF-10A acini (data not shown). These fully formed acini were then treated with diluent or 100 nM 4-HT for 5 days. To simplify interpretation, exogenous epidermal growth factor (EGF), which is normally present at 1 ng/ml in organotypic culture growth medium, was omitted from the medium at the time of treatment with 4-HT in all experiments.

**Figure 1:**
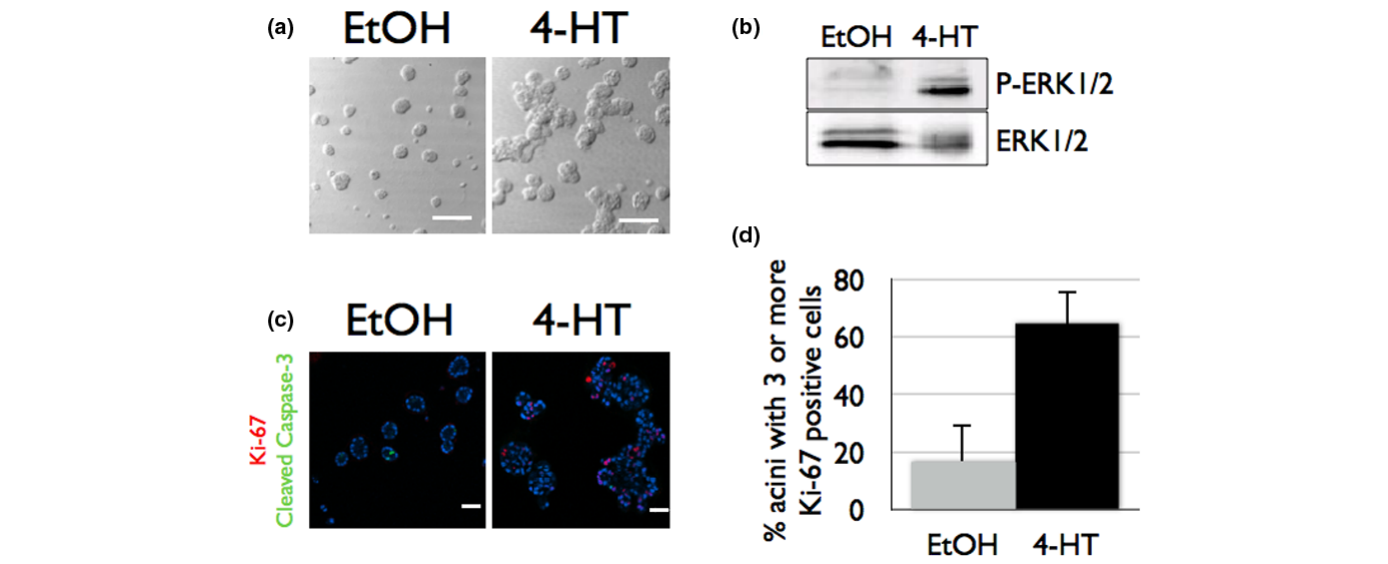
Activation of Raf:ER induces pre-invasive epithelial growth in cultured acini. (**a) **Raf:ER-MCF-10A cells plated for three-dimensional culture were grown for 10 days. At day 10, diluent or 100 nM 4-hydroxytamoxifen (4-HT) was added and cultures were grown for an additional 5 days. Differential interference contrast images are shown. Bars = 150 μM. (**b**) The lysates of day 10 acini treated for 48 hours with diluent or 100 nM 4-HT were immunoblotted with α-phospho-ERK1/2 (top) and α-ERK1/2 antibodies. (**c**) Raf:ER-MCF-10A cells were grown as described in (a). Confocal cross-sections of acini immunostained with α-Ki-67 (green) and α-cleaved caspase 3 (red) and counterstained with Hoechst (blue) are shown. Bars = 50 μm. (**d**) The percentage of acini containing three or more Ki-67 cells was quantified. Data are the mean ± standard error of the mean of 100 acini counted in three independent experiments.

Acini treated with 4-HT at day 12 lost their spherical shape and were larger then control acini (Figure 1a), as judged by differential interference contrast microscopy. Raf:ER expression was typically increased in at least 90% of cells within an individual acinar structure 48 hours after administration of 4-HT (data not shown), and the induction of Raf:ER promoted a large increase in the level of activated ERK1/2 (Figure 1b). Examination of the arrangement of cells, as judged by the position of nuclei and appearance under differential interference contrast microscopy, revealed a loss of spherical architecture and of cells occupying the lumens of acini (Figure 1a,c), consistent with our previous findings [25].

To determine the frequency with which Raf:ER activation increases cell proliferation, acini treated with 4-HT for 48 hours were fixed and immunostained with an antibody towards Ki-67, a marker of proliferation. Only 17% of the control acini contained three or more cells expressing Ki-67, whereas 65% of the acini treated with 4-HT had three or more cells expressing Ki-67, indicating that the activation of ERK1/2 is sufficient to stimulate an increased rate of proliferation in cultured acini (Figure 1c,d).

A key step in the development of breast cancer is survival of cells in the luminal space [3]. Previous studies have demonstrated that normal cells in the lumen undergo caspase-dependent apoptosis as indicated by positive staining for the cleaved and activated forms of caspase 3 and caspase 9 [24]. We found that, unlike control acini, Raf:ER-expressing MCF-10A acini had few if any cleaved caspase-3-containing cells in their lumens, indicating that these cells were resistant to apoptosis (Figure 1c). Collectively, these results demonstrate that the activation of Raf:ER in differentiated epithelium induces an expansion of acinar size and filling of the luminal space through the coordination activation of both proliferative and prosurvival signaling pathways in organotypic culture.

### Raf:ER does not require autocrine activation of EGFR to promote the disruption of epithelial architecture

The characterization of Raf–MEK1/2–ERK1/2 signaling in two-dimensional culture systems has suggested a predominant role for the autocrine activation of EGFR in ERK1/2-driven proliferation and cell survival [30,31]. Considering ERK1/2 are active in epithelial cancers, including breast cancer, if ERK1/2 requires autocrine activation of EGFR, than the therapeutic blockade of EGFR will block ERK1/2-driven tumorigenic responses. Determining the contribution of EGFR to ERK1/2-driven pre-invasive mammary epithelial cell growth is therefore critical considering the current clinical trials investigating therapeutic inhibitors of EGFR [32].

**Figure 2:**
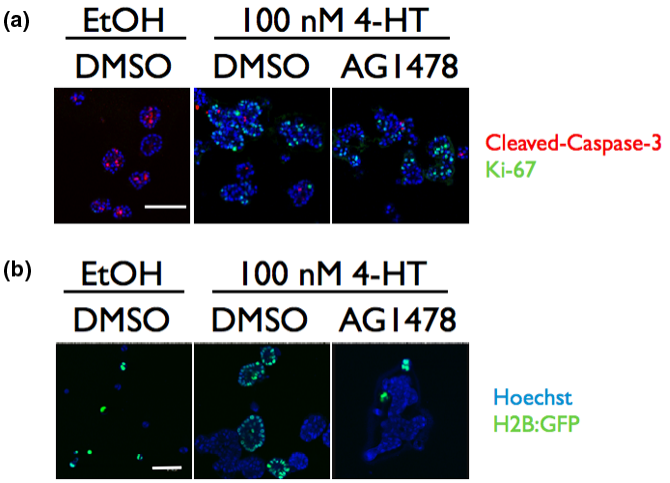
Autocrine epidermal growth factor receptor activation unnecessary for ERK1/2 stimulation of proliferation or apoptosis resistance. **(a) **Acini were grown for 10 days and then treated with diluent, with 100 nM 4-hydroxytamoxifen (4-HT) or with 100 nM 4-HT + 300 nM AG1478 (epidermal growth factor receptor (EGFR) inhibitor) and were immunostained with α-Ki-67 (green) and α-cleaved caspase 3 (red) antibodies and counterstained with Hoechst (blue). The acini shown are representative of 100 acini observed in three independent experiments. No more then 10 acini in the 4-HT-treated acini or 4-HT + AG1478-treated acini had greater then 10% of cells in the lumen stain positive for cleaved caspase 3 in any of the experiments. Bar = 75 μm. DMSO, dimethylsulfoxide. **(b) **Raf:ER-expressing and H2B:GFP-expressing MCF-10A cells were plated at a 1:1 ratio and grown in three-dimensional culture for 13 days with diluent, with 100 nM 4-HT in the absence of EGF, or with 100 nM 4-HT, no EGF and 300 nM AG1478 (EGFR inhibitor). The nuclei of Raf:ER acini are seen with Hoechst (blue) and the nuclei of the H2B:GFP-MCF-10A cells are visualized with green fluorescence protein (GFP) (green) overlaying Hoechst (blue). Bar = 50 μm.

We tested whether autocrine EGFR activation was necessary for proliferation in organotypic culture using the pharmacological EGFR kinase inhibitor AG1478. We found that inhibiting EGFR activity with 300 nM AG1478 had no effect on the Raf:ER-induced disruption of epithelial architecture or stimulation of proliferation as judged by Ki-67 staining (Figure 2a). It has been suggested that cells in the lumens of acini undergo anoikis due to their inability to interact with basement membrane [24]. Resistance to anoikis in Raf:ER-MCF-10A cells requires activation of EGFR, so we examined whether EGFR activation is necessary for survival of cells in the lumens of Raf:ER-induced acini [33]. Blockade of EGFR kinase activity with AG1478 did not cause caspase-dependent apoptosis in lumens of Raf:ER-induced acini as judged by immunostaining for cleaved caspase 3 (Figure 2a).

We next determined whether ERK1/2 activation induces the production of autocrine growth factors in organotypic culture. Since the growth of MCF-10A cells in organotypic culture is absolutely dependent on EGF [21], we reasoned that if Raf:ER-induced acini are producing autocrine EGFR agonists, then Raf:ER-induced acini could support the growth of wild-type MCF-10A cells cultured in the absence of exogenous EGF [18]. To distinguish wild-type MCF-10A cells from the Raf:ER-MCF-10A cells, we generated a wild-type MCF-10A cell line that stably expressed the H2B-GFP fusion protein. Raf:ER cells were co-cultured with MCF-10A-H2B:GFP cells at a 1:1 plating ratio. The cultures were grown with diluent or 100 nM 4-HT in the absence of EGF for 13 days. In the control cultures treated with diluent, neither Raf:ER cells nor the MCF-10A-H2B:GFP cells proliferated to form acini. On the other hand, when Raf:ER was activated by 100 nM 4-HT, both the Raf:ER cells and the MCF-10A-H2B:GFP cells grew to form acini (Figure 2b). Over 85% of Raf:ER and MCF-10A/H2B:GFP cells grew to acini of at least 30 μM in diameter.

The acini are not mixed groups of cells, because acini are entirely formed from cells that express H2B:GFP or from cells that do not (Figure 2b). The ability of acini expressing activated Raf:ER to promote growth of co-cultured normal MCF-10A acini in the absence of EGF indicates that activated Raf:ER acini secrete autocrine growth factors that complement the absence of EGF. We confirmed that the growth-promoting autocrine growth factors were acting on EGFR by growing the co-cultures in the presence of 300 nM AG1478 (Figure 2b). Only one or two acini out of 100 MCF-10A/H2B:GFP cells counted grew larger than five cells in three independent experiments. Activation of ERK1/2 in differentiated mammary epithelium does indeed therefore induce the production of autocrine growth factors that act on EGFR. One candidate factor is heparin-binding EGF [30].

### Raf:ER activation promotes the induction of c-Fos and the decreased expression of Bim

**Figure 3:**
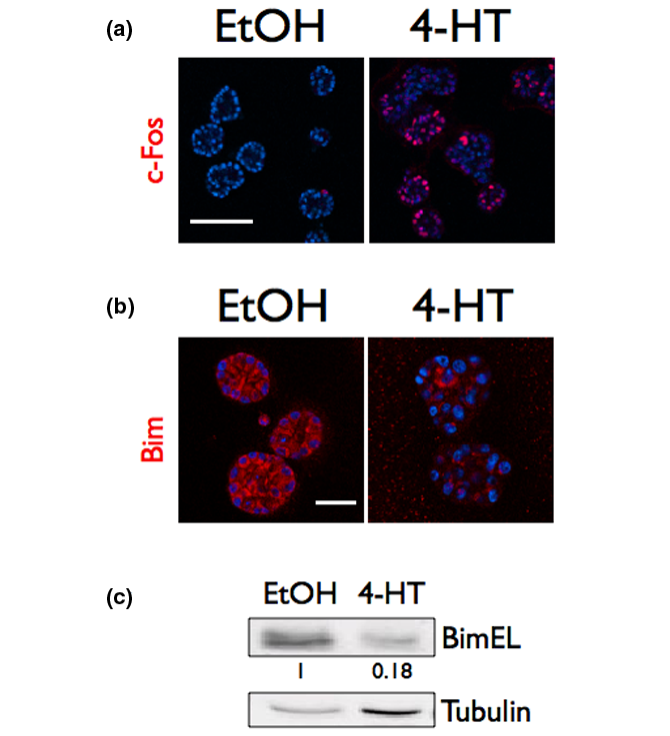
Raf:ER activates multiple downstream effectors. Raf:ER three-dimensional cultures were grown for 10 days, treated with diluent or 100 nM 4-hydroxytamoxifen (4-HT) and grown for an additional 48 hours. (**a**) Acini were immunostained with α-c-Fos (red) and counterstained with Hoechst (blue). Bar = 150 μm. (**b**) Acini were immunostained with α-Bim antibody (red) and counterstained with Hoechst (blue). Bar = 30 μm. (**c**) The lysates of acini were immunoblotted with α-Bim antibody (upper panel) or α-α-tubulin (bottom panel) antibodies. The Bim signal intensity from the 4-HT-treated acini normalized to the tubulin signal intensity and divided by the normalized Bim signal intensity from the ethanol-treated acini is shown.

We next explored the intracellular targets of ERK1/2 that promote proliferation and cell survival. Immediate early gene products, such as the transcription factor c-Fos, regulate cell proliferation in a variety of cell types [34]. ERK1/2 can enhance c-Fos expression through indirect regulation of c-*fos *transcription and phosphorylation-dependent stabilization of c-Fos protein [35]. Whether c-Fos expression is elevated in response to ERK1/2 activation or any oncogenic stimuli in differentiated epithelium in organotypic culture is not known. We examined c-Fos expression in day 10 acini or later acini after treatment with 100 nM 4-HT for 48 hours by immunostaining, and found that c-Fos protein levels were increased in acini treated with 100 nM 4-HT (Figure 3a). The elevated expression of c-Fos suggests that ERK1/2-stimulated proliferation could in part be regulated by c-Fos. The single-cell-level analysis provided by our immunofluorescence analysis also demonstrates that c-Fos expression does not directly correlate with the degree of disruption of epithelial architecture (Figure 3a). This indicates that the variations in epithelial phenotype that are observed are not simply due to differences in the level of c-Fos expression, and demonstrates the complexity of intracellular biochemical signaling involved in stimulating pre-invasive growth in organotypic culture.

When cells occupy the lumens of MCF-10A acini, cell survival cues provided by integrin contacts with the basement membrane are lost. The intracellular signaling architecture of epithelial cells must therefore be altered for cells to survive in the luminal space. The expression level of the protein proapoptotic BH3 domain-containing protein Bim is incrementally increased in all of the MCF-10A cells as they differentiate and form acini in organotypic culture [36]. This apoptotic trigger is counterbalanced by unknown biochemical signals stimulated by cell attachment to the surrounding basement membrane [36]. Reduced expression of Bim is sufficient to delay apoptosis of cells in lumens of MCF-10A acini and the developing mammary gland, which suggests that the differentiation-dependent increase in Bim expression triggers apoptosis of centrally located cells and formation of a lumen [36,37]. Stable expression of a constitutively active form of MEK1 is sufficient to reduce Bim expression in MCF-10A acini, and Raf:ER induction can reduce Bim expression in MCF-10A cells in monolayer culture and in detached cells [33,36]. The sufficiency of acute ERK1/2 activation to reduce Bim expression in differentiated mammary epithelium, however, has not been tested. We examined Bim expression 48 hours after Raf:ER activation by immunostaining and immunoblotting, and found the Bim expression level was indeed decreased (Figure 3b,c). This result suggests that Raf:ER activation promotes resistance to apoptosis and the occupation of the lumen by mammary epithelial cells in part through decreasing the expression level of Bim.

### Raf:ER activation of AKT promotes degradation of p27 and cell cycle progression in mammary organotypic culture

Previous studies in two-dimensional culture models have shown that Raf:ER indirectly stimulates the phosphorylation of the AGC kinase AKT on serine 473 [30]. Overexpression of AKT1 is sufficient to delay MCF-10A growth arrest in three-dimensional culture and cooperates with overexpressed cyclin D_1 _or the viral oncoprotein HPV E7 to promote proliferation [18]. AKT also regulates proliferation in malignant T4-2 mammary epithelial cells in three-dimensional culture [38].

**Figure 4:**
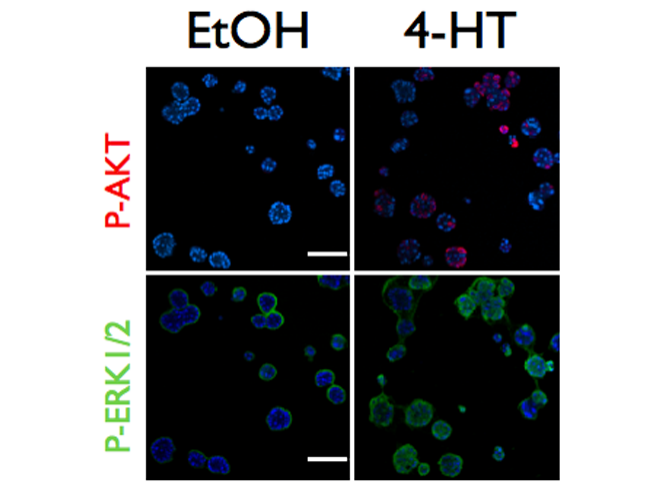
Raf:ER activates AKT in organotypic culture. Raf:ER three-dimensional cultures were grown for 10 days, treated with diluent or 100 nM 4-hydroxytamoxifen (4-HT) and grown for an additional 48 hours. Acini were immunostained with both α-phospho-AKT^S473 ^(red, top panels) and α-phospho-ERK1/2 (green, bottom panels) and counterstained with Hoechst (blue). The same acini are shown in the upper and lower panels of each treatment condition to demonstrate the relationship between ERK1/2 activation and AKT phosphorylation. There is some nonspecific staining of the Matrigel surrounding acini in the phospho-ERK1/2 images. Bars = 150 μm.

Considering the potential role of AKT signaling in the disruption of epithelial architecture induce by Raf:ER, we examined the activation state of AKT using an antibody that recognizes AKT phosphorylated at serine 473 by immunostaining. We found that Raf:ER activation increases the fraction of the cells that immunostain positive for phospho-Ser473 AKT (Figure 4, upper panels). The stochastic nature of AKT phosphorylation we observed is consistent with the pattern of AKT phosphorylation in normal MCF-10A acini earlier in their development [21]. Consistent with increased Raf:ER expression being observed in the majority of cells in an acinus, the majority of cells stained positive for phospho-ERK1/2 (Figure 4, lower panels). Although AKT phosphorylation occurred exclusively in acini where phosphorylated ERK1/2 was detected (Figure 4), however, double staining for phospho-ERK and phospho-AKT showed that activated Akt was only present in a fraction of cells with activated ERK. The stochastic pattern of AKT serine 473 phosphorylation is therefore unlikely to be due to variations in Raf:ER expression or ERK1/2 activity, but it does depend on ERK activation. We did not detect phospho-Ser473-AKT until 24 hours after Raf:ER activation, whereas increased expression of c-Fos and phosphorylation of p90 ribosomal S6 kinase (p90RSK), a direct target of ERK1/2, were first observed 2 hours after 4-HT treatment (data not shown).

These collective results suggest that ERK1/2 regulation of AKT is indirect. Whether AKT phosphorylation is observed only in a small fraction of cells because AKT is phosphorylated and dephosphorylated in an oscillatory fashion, or whether there are variations in the strength of autocrine/paracrine stimulation leading to AKT activation, is not known.

### Raf:ER-induced disruption of epithelial architecture requires phosphoinositide-3 kinase activity

**Figure 5:**
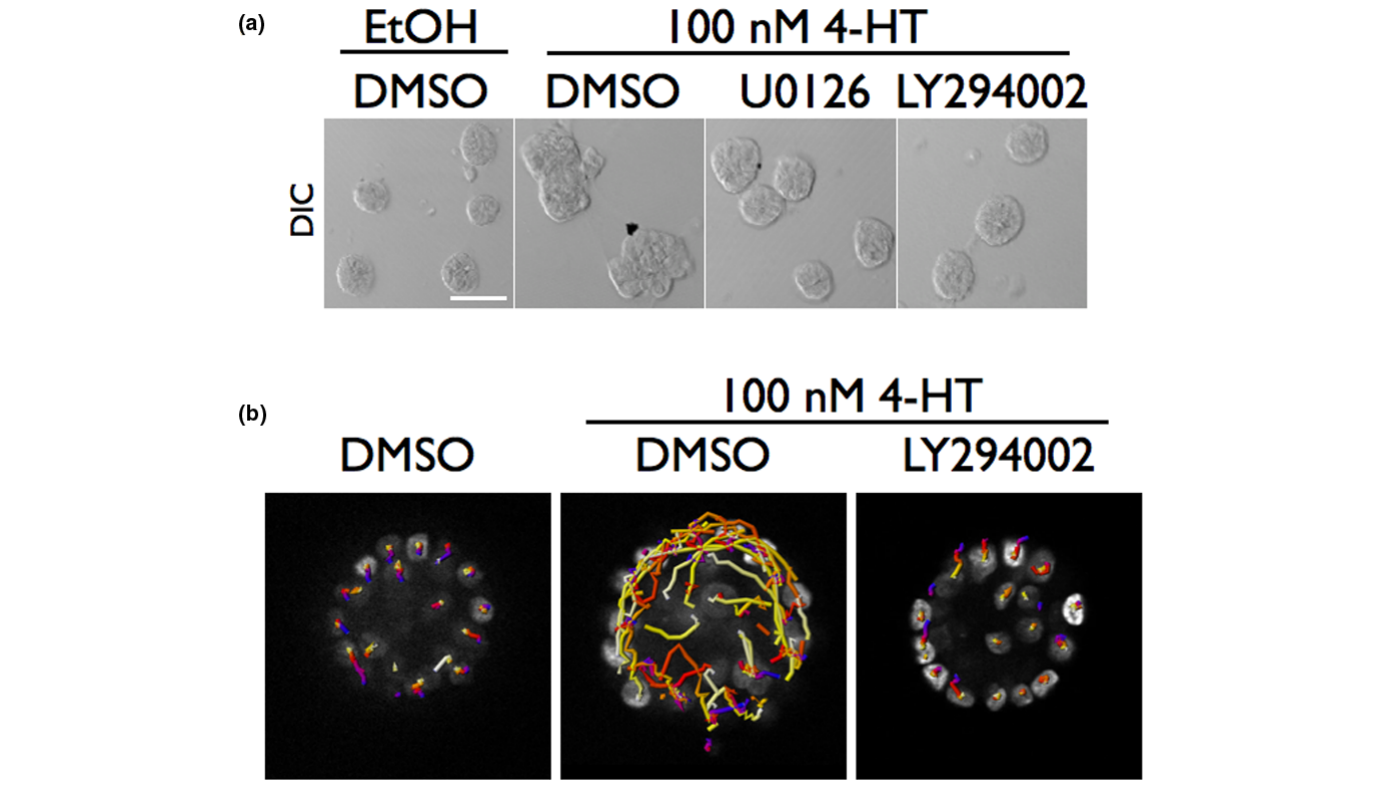
Phosphoinositide-3 kinase activity is necessary for the disruption of epithelial architecture. **(a) **Acini were grown for 10 days and then treated with diluent, 100 nM 4-hydroxytamoxifen (4-HT) or 100 nM 4-HT and inhibitor. U0126 (10 μM) and LY294002 (50 μM) were used. Fresh media with diluent, with 4-HT or with 4-HT and inhibitor was added after 24 hours primary treatment, and acini were cultured for another 24 hours (48 hours total treatment time). Bar = 150 μM. DIC, differential interference contrast microscopy; DMSO, dimethylsulfoxide. **(b) **Day 10 Raf:ER-H2B:GFP acini were treated with diluent, 100 nM 4-HT or 100 nM 4-HT and 50 μM LY294002 (phosphoinositide-3 kinase inhibitor) for 24 hours and then imaged for 20 hours at 30-minute intervals. The total movement of the cells of a representative acinus from each condition from three independent experiments is shown. The H2B:GFP-labeled nuclei are at white and are located at their respective positions at the end of the 20 hours of imaging. Colored scale bar represents increasing time. Results shown are representative of at least 10 acini imaged per condition in three independent experiments.

It is likely that the induction of Raf:ER leads to phosphoinositide-3 kinase (PI-3K) activation, since it is known that PI-3K activity is required for phosphorylation of AKT serine 473. We therefore next set out to determine the relative importance of MEK1/2–ERK1/2 and PI-3K signaling in stimulating the phenotypes observed in Raf:ER-induced acini using pharmacological inhibitors. Cells were grown for 10 days or more and were treated with 100 nM 4-HT for 48 hours with or without the inhibitor. As expected, inhibition of MEK1/2 with 10 μM U0126 prevented any gross change in acinar morphology (Figure 5a). Blockade of PI-3K with 50 μM LY294002 also prevented Raf:ER-induced morphological changes (Figure 5a, upper panels). These results suggest that PI-3K activity is required for the disruption of mammary epithelial architecture induced by Raf:ER activation.

As discussed above, we have previously developed a method for imaging cells in Raf:ER-induced acini at single-cell resolution through imaging a histone–GFP fusion protein, H2B-GFP. Using this unbiased discovery approach we have found that Raf:ER activation induces a non-invasive form of motility that promotes the disruption of epithelial architecture. How cells become motile in response to either ERK1/2 activation or prior to invasion is not known. Defining both how ERK1/2 activation induces movement and also how movement is induced in multicellular epithelial acini is necessary to understand how cells become motile and invasive during breast cancer progression. Raf:ER acini were grown for 10 or days more in organotypic culture and the acini were stimulated with 100 nM 4-HT in the presence or absence of the PI-3K inhibitor LY294002. We found that the treatment of acini with LY294002 was sufficient to block the induction of noninvasive motility in all of the acini that were stimulated by Raf:ER activation (Figure 5b and see Additional files 1 to 3). In contrast, over 50% of the Raf:ER-induced acini contained five or more motile cells under these conditions. These results demonstrate that the disruption of epithelial architecture induced by Raf:ER requires differentiated mammary epithelial cells to integrate signals from both ERK1/2 and PI-3K. This is the first demonstration that PI-3K activity is necessary for motility in mammary epithelial acini or in response to ERK1/2 activation.

### PI-3K activity is not necessary for reduced cell–cell adhesion or the induction of MLC2 phosphorylation by ERK1/2

**Figure 6:**
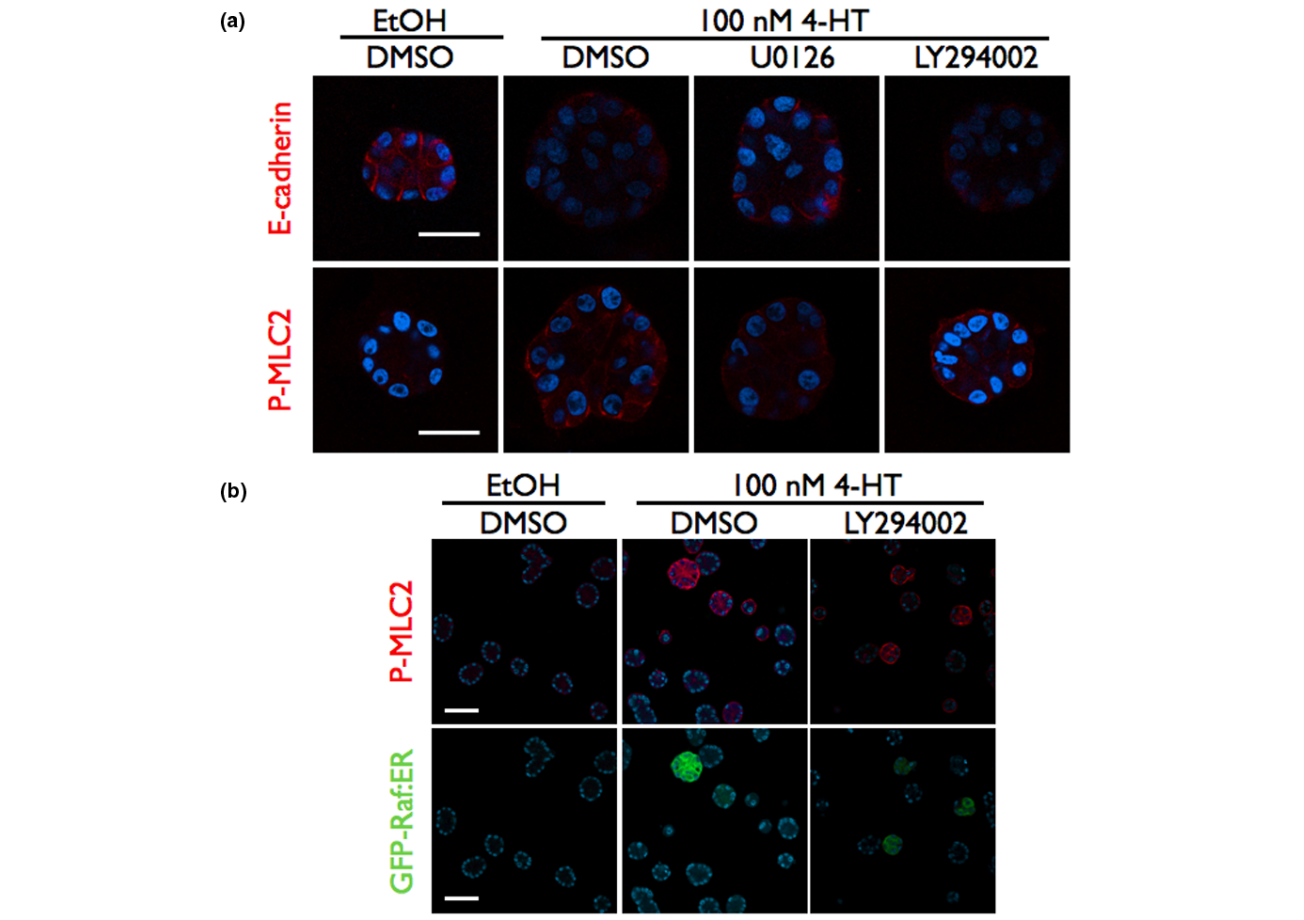
Phosphoinositide-3 kinase activity is not necessary for reduced cell–cell adhesion or MLC2 (Ser-19) phosphorylation. **(a) **Raf:ER acini were treated with diluent, with 100 nM 4-hydroxytamoxifen (4-HT) or with 100 nM 4-HT and inhibitor as indicated after at least 10 days of growth in organotypic culture. Acini were immunostained with either anti-E-cadherin (red, top panels) or anti-phospho-MLC2 (Ser-19) (red, bottom panels) antibodies and counterstained with Hoecsht (blue, nuclei). Results are representative of at least three independent experiments. Bar = 30 μm. DMSO, dimethylsulfoxide. **(b) **Green fluorescence protein (GFP)–Raf:ER acini were cultured as described in (a). Acini were immunostained with anti-phospho-MLC2 (Ser-19) antibody (red, top panels) and counterstained with Hoecsht (blue). The expression of GFP-Raf:ER (green) is shown in the bottom panels. Bar = 50 μm.

We next investigated the molecular basis for the requirement of PI-3K activity in the induction of cell motility. We have shown previously that Raf:ER activation induces cells to move independently of each other, and that this independent movement correlates with the loss of E-cadherin at cell–cell contacts [25]. We examined whether PI-3K activity was necessary for the loss of E-cadherin induced by Raf:ER, and found that treatment of acini with LY294002 had no effect on the loss of E-cadherin at cell–cell contacts (Figure 6a, upper panels).

The induction of non-invasive motility in response to Raf:ER activation requires the phosphorylation of MLC2 in a Rho kinase-dependent and myosin light-chain kinase-dependent manner [25]. The pharmacological blockade of PI-3K activity prevents RhoA and Rho kinase activation in neutrophil-like HL-60 cells [39], which suggested to us that the inhibition of PI-3K could be reducing the level of MLC2 phosphorylation and contraction in the Raf:ER-induced acini. We treated day 10 acini with diluent or LY294002 at the time of Raf:ER activation and examined the MLC2 phosphorylation at Ser19 using a phoshospecific antibody. The treatment of acini with LY294002 did not reduce MLC2 phosphorylation at Ser19 in response to Raf:ER activation or GFP-Raf:ER activation under conditions where AKT phosphorylation is reduced (Figure 6a, lower panels, and 6b). Only of a subset of acini show GFP–Raf:ER expression because the cell line did not undergo drug selection to select for GFP–Raf:ER. Also, GFP–Raf:ER expression is increased after treatment with 4-HT because of increased protein stability [29]. Our results indicate that PI-3K is necessary for at least one more additional step for cells to become motile since PI-3K activity is not required for either the reduction of E-cadherin expression or for the phosphorylation of MLC2 on Ser19.

### ERK1/2 activation of AKT correlates with reduced p27 expression

**Figure 7:**
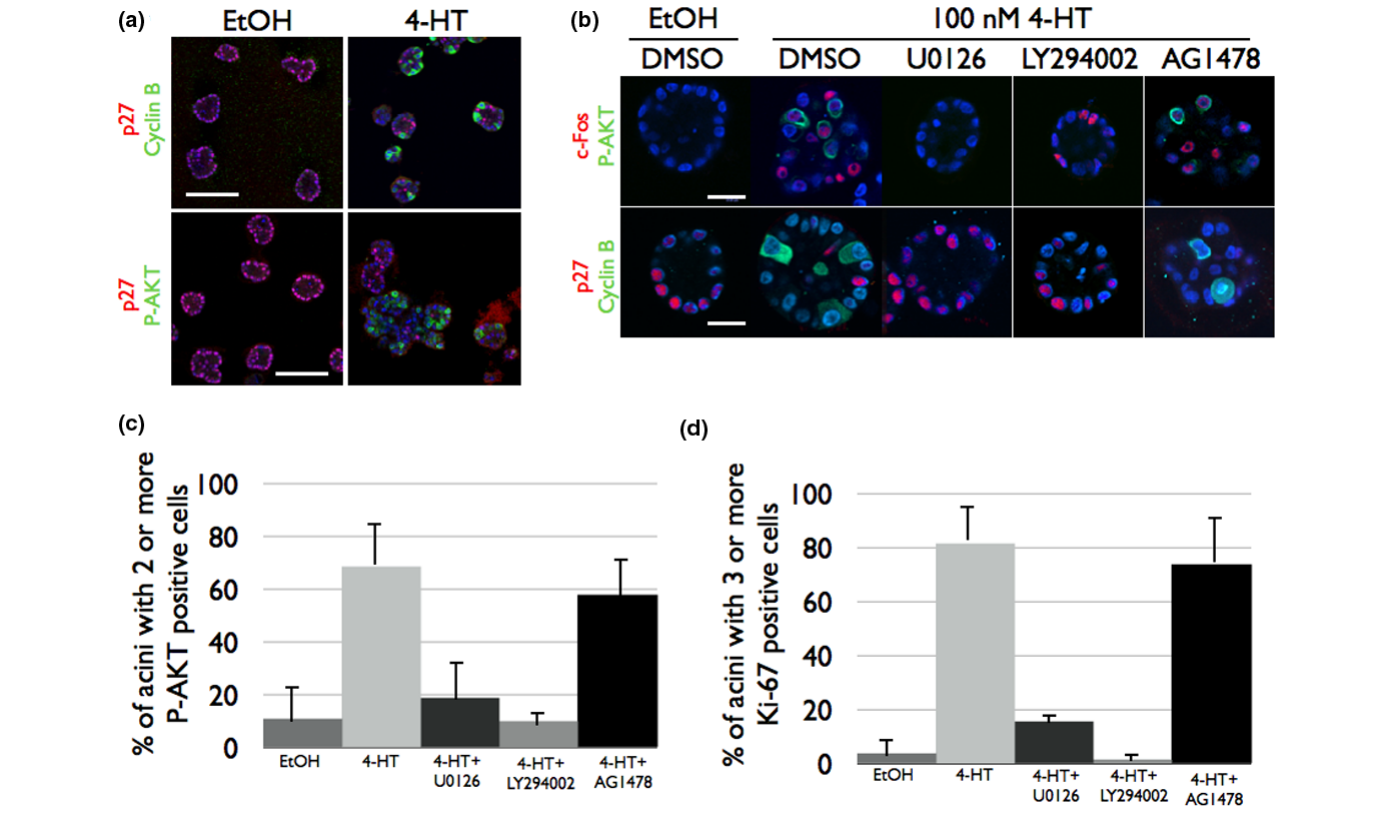
Cell cycle progression in Raf:ER-stimulated acini requires phosphoinositide-3 kinase activity. (**a**) (Top panels) Acini were immunostained with α-cyclin B_1 _(green) or α-p27 (red) and counterstained with Hoechst (blue). (Bottom panels) Acini were immunostained with α-phospho-AKT^S473 ^(green) or α-p27 (red) and counterstained with Hoechst (blue). (**b) **Acini were grown for 10 days and then treated with diluent, with 100 nM 4-hydroxytamoxifen (4-HT) or with 100 nM 4-HT and inhibitor. U0126 (10 μM), LY294002 (50 μM) and AG1478 (300 nM) were used. Fresh media with, diluent, with 4-HT or with 4-HT and inhibitor was added after 24 hours of primary treatment, and acini were cultured for another 24 hours (48 hours total treatment time). (Upper panels) α-c-Fos (red) and α-phospho-AKT^S473 ^(green). (Lower panels) α-p27 (red) and α-cyclin B (green). All samples were counterstained with Hoechst (blue). Bar = 30 μm. DMSO, dimethylsulfoxide. (**c**) The number of acini containing at least two phospho-AKT^S473^-positive cells was scored. Data are the mean ± standard error of the mean of 100 acini scored in three independent experiments. (**d**) The percentage of acini containing three or more Ki-67 cells was quantified. Data are the mean ± standard error of the mean of 100 acini counted in three independent experiments.

Real-time imaging showed that cells in Raf:ER-induced acini did not divide when they were treated with LY294002. Consistent with this observation, the substantial increase in the number of acini containing two or more cells with phospho-AKT (Figure 4, upper panels) suggested a role for AKT in cell proliferation in organotypic culture. The transition from G_1 _into the S phase of the cell cycle requires a reduction in the expression of the Cdk inhibitor protein p27 [40], which in part is regulated by AKT [41]. Failure to suppress p27 expression prevents expression of cyclin B_1 _and activation of Cdk1 [42]. Acini expressing activated Raf:ER had few if any cells expressing p27 but contained a number of cells expressing cyclin B_1 _(Figure 7a, upper panels). Because we can examine biochemical signal transduction pathways at single cell resolution, we were able to directly compare the activation state of AKT with the expression of p27. We found an inverse correlation between AKT activation and p27 expression, as p27 was not detected in any cells containing detectable levels of phospho-AKT (Figure 7a, lower panels). This result strongly suggests that AKT stimulates cell cycle progression by suppressing the expression of p27 in our model.

### PI-3K activity is necessary for Raf:ER-stimulated p27 degradation and cyclin B induction

To determine whether PI-3K and AKT activity was indeed required for proliferation, day 10 acini or later acini were treated with 100 nM 4-HT for 48 hours with or without inhibitor. Inhibiting MEK1/2 or PI-3K was sufficient to prevent AKT activation, the suppression of p27 expression, and cyclin B_1 _induction (Figure 7b).

In monolayer culture, autocrine EGFR activation is necessary to activate AKT [30], so we determined whether autocrine EGFR activation is necessary for AKT activation in organotypic culture. EGFR activity was not necessary for activation of AKT in 4-HT-treated Raf:ER acini, however, and consequently AG1478 had no effect on the suppression of p27 and cyclin B_1 _induction (Figure 7b,c). Furthermore, EGFR inhibition was also ineffective compared with either MEK1/2 or PI-3K blockade at reducing proliferation as judged by Ki-67 expression (Figure 7d).

Since the concentration of AG1478 used blocked the growth of co-cultured MCF-10A cells (Figure 2b), the failure of AG1478 to block AKT phosphorylation, p27 degradation or Ki-67 expression was probably not due to a failure to inhibit EGFR.

These results demonstrate that the PI-3K–AKT signaling pathway is necessary for ERK1/2 signaling to stimulate proliferation in differentiated mammary epithelial acini.

## Discussion

We have demonstrated that the persistent activation of the Raf–MEK1/2–ERK1/2 mitogen-activated protein kinase module promotes the development of pre-invasive mammary lesions from differentiated epithelium in organotypic culture. This finding indicates that persistent ERK1/2 activation in luminal epithelial cells might contribute to the development of mammary tumors. It is known that ERK1/2 is activated by oncogenes, such as ErbB2; however, our results demonstrate that persistent activation of ERK1/2 can induce growth and survival in the absence of receptor tyrosine kinase mutation or overexpression. It is possible that unidentified genetic abnormalities, or combinations of abnormalities, promote activation of ERK1/2 in mammary epithelium. This conclusion is supported by the observation that persistent ERK1/2 activation is found in a wide range of patient-derived mammary tumor cell lines, many of which do not harbor amplified expression of ErbB2 [43] and the sequencing of breast cancer tumor genomes [5]. Furthermore, by uncoupling the activation of the Raf–MEK1/2–ERK1/2 module from a specific oncogenic lesion, our results suggest that the inappropriate expression of growth factor receptor ligands could promote tumorigenesis through the sustained stimulation of ERK1/2.

The number of ductal carcinoma *in situ *(DCIS) cases identified in the United States annually has risen from 4,800 in 1983 to over 50,000 today [4]. After identification, DCIS lesions are surgically removed with a breast-conserving excision and patients may undergo either a course of adjuvant therapy targeted to block the action of the hormone estrogen or receive gamma irradiation to kill the remaining proliferating tumor cells [4]. The risk of a recurrent growth developing 15 years after lumpectomy is between 16 and 19%, and thus patients are required to undergo continual surveillance [44]. One-half of recurrent growths are invasive breast cancer, which is more difficult treat and pose a much greater threat of metastasis [44-46]. It is likely that early-stage epithelial tumors, such as DCIS, are susceptible to new and more efficacious diagnostic tests and forms of therapy. Our results demonstrate that ERK1/2 activation is sufficient to promote proliferation and cell survival in the lumens of mammary epithelial acini, which are characteristic behaviors required for recurrent tumor growth after lumpectomy.

These findings warrant further investigation of the activity level of the ERK1/2 signaling pathway in patient samples to determine the frequency of ERK1/2 activation in early-stage breast cancer and whether there is a correlation between ERK1/2 activation and recurrent growth after lumpectomy. In the event that a positive connection between ERK1/2 activation and recurrent growth is revealed, there are a number of inhibitors of MEK1/2, the direct upstream activators of ERK1/2, that have undergone various stages of in clinical testing and could be tested as adjuvant therapy in the clinic [47].

### Bim and c-Fos of targets of ERK1/2 signaling in differentiated mammary epithelial acini

We have identified c-Fos and Bim as downstream effectors of ERK1/2 that can contribute to the proliferation and survival of differentiated mammary epithelial cells in the lumens of epithelial acini. These targets of ERK1/2 signaling are worthy of investigation in patient samples to determine whether ERK1/2 signaling promotes early-stage human breast cancer progression through similar mechanisms to those observed in organotypic culture.

In addition to promoting c-Fos expression and Bim degradation, ERK1/2 directly phosphorylates a vast array of proteins that are also likely to contribute to the observed phenotypes. For instance, p90 RSK1/2 are activated by direct ERK phosphorylation on serine 363, in the linker between the N-terminal and C-terminal catalytic domains, and threonine 573, in the activation loop of the C-terminal catalytic domain, resulting in autophosphorylation at serine 380 and creation of a docking site for PDK1, which then phosphorylates serine 239 [48,49]. Once activated, p90 RSK1/2 promotes transcription through direct phosphorylation of transcription factors including the serum response factor and c-Fos [50-52]. The transcriptional co-activator CREB binding protein is also a target for p90 RSK [53]. Furthermore, p90 RSK can promote cell survival through the phosphorylation and inactivation of the Bcl-2-associated death promoter protein and the activation of the mammalian target of rapamycin protein by phosphorylating and inactivating tuberous sclerosis complex 2 [54,55].

This is just one of many examples of the molecular mechanisms by which ERK1/2 can promote pre-invasive tumor growth. The identification of the ERK1/2 substrates that are required to promote cell growth and survival will further provide a molecular framework with which to understand pre-invasive tumor development.

### PI-3K activity is necessary for ERK1/2-stimulated proliferation

We have shown that the persistent activation of ERK1/2 increases the activity of the parallel PI-3K–AKT signaling module, but in a stochastic manner in cells within an acinus. The activity of the PI-3K, and possibly AKT, is necessary for the progression of MCF-10A cells through the cell cycle, as has been previously demonstrated in fibroblasts [42]. The identity of the signaling circuit connecting ERK1/2 to PI-3K in epithelial organotypic culture is not known. Interestingly, autocrine activation of EGFR was not necessary for AKT activation in our organotypic culture model, which is in contrast to results that were obtained when Raf:ER was induced in MCF-10A cells grown as two-dimensional monolayers [30]. This discrepancy could be due to subtle variations between MCF-10A cell lines or differences in the expression level of the Raf:ER protein. Alternatively, a distinct mechanism by which ERK1/2 signaling activates PI-3K could be present in organotypic culture, and possibly *in vivo*. For example, although EGFR activation *per se *is not necessary for proliferation of Raf:ER-induced acini, we do not rule out a role for autocrine growth factors in Raf:ER-stimulated proliferation or PI-3K activation in organotypic culture. This is because Raf:ER activation promotes the autocrine production of FGF-2 and VEGF, which act on non-EGFR receptor tyrosine kinases, and of heparin-binding EGF, which can elicit heterodimerization of ErbB4 with ErbB2 [30]. Each of these factors activates receptors or receptor combinations that are capable of activating PI-3K, and thus one or more of these autocrine ligands could promote the phosphorylation and activation of PI-3K and AKT in our model.

### PI-3K activity is necessary for ERK-stimulated motility

Our understanding of how cells become motile in response to ERK1/2 activation is limited. ERK1/2 can phosphorylate myosin light-chain kinase to promote myosin contraction and can also phosphorylate calpain to promote the severing of integrin attachment to substratum in fibroblasts [56,57]. We have shown that ERK1/2 promotes MLC2 phosphorylation through myosin light-chain kinase in mammary epithelial acini; however, a pharmacological inhibitor of calpain has had no effect on cell motility in our model (GW Pearson, unpublished observation). The targets of ERK1/2 signaling that regulate cell motility in general or in mammary epithelial acini are therefore a mystery. We have discovered that PI-3K signaling is upregulated by ERK1/2, and that PI-3K activity is necessary for cell motility in mammary epithelial acini. Although PI-3K and the phospholipid products of PI-3K activity can be elevated through mutation of the catalytic domain of PI-3K or deletion of the phosphatase and tensin homolog lipid phosphatase or amplification and activation of transmembrane receptor proteins, the activation of PI-3K in breast cancer does not require these mutagenic events [58-60]. It is then possible that ERK1/2 activity could drive cell movement, in part, through the activation of PI-3K in some breast cancers.

### PI-3K activity is necessary for cell motility in mammary epithelial acini

How cells become motile in mammary epithelial acini is not well understood. We have recently determined that cells can become motile in the absence of invasion [25]. This finding has potential clinical relevance, because motile cells could be present in pre-invasive lesions, such as DCIS, and thus portend a greater risk of future invasive growth. Whether there are indeed motile cells in pre-invasive lesions is not yet known. A step towards determining how cells become motile during tumorigenesis is the identification of the intracellular signaling pathways that are necessary or sufficient to induce cell movement in these multicellular structures. We have already found that ERK1/2 activation is sufficient to induce movement and that this ERK1/2-driven motility requires MLC2 phosphorylation and a reduction in E-cadherin expression [25]. We have now determined that PI-3K activity is necessary for the induction of motility induced by ERK1/2 signaling in mammary epithelial acini.

The requirement of PI-3K activity for Raf:ER-stimulated cell motility is independent of MLC2 phosphorylation or E-cadherin expression, which suggests that PI-3K regulates at least one additional process that is necessary for cells to become motile in mammary epithelial acini. PI-3K signaling has been extensively studied in the regulation of chemotaxis in the slime mold *Dictyostelium *and neutrophils [61]. In these model systems, PI-3K contributes the production of phosphatidylinositol (3,4,5)-triphosphate at the leasing edge of the cell, which is necessary for the polarization of the cell and the directional migration towards a chemoattractant [61]. PI-3K activity is necessary for the chemotaxis of additional cell types, including some patient-derived breast cancer cell lines, possibly through an analogous mechanism [62]. Whether cells in epithelial acini are moving by chemotaxis is not known. In fact, cells move in different directions within an acinus – which suggests that chemotaxis, and by extension a requirement for sustained polarization of cells, is not necessary for the movement observed. Considering this possibility, PI-3K activity probably regulates motility in mammary epithelial acini through a mechanism distinct from the polarization necessary for chemotaxis observed in other model systems. In the future, determining how PI-3K regulates movement in mammary epithelial acini will serve to further explain how cells become motile during breast cancer progression.

## Conclusions

Our results demonstrate that the activation of the Raf–MEK1/2–ERK1/2 mitogen-activated protein kinase module is sufficient to induce cell proliferation, survival and motility in cultured mammary epithelial acini. In addition, PI-3K activity was required for proliferation and survival induced by ERK1/2 activation. Each of these cell behaviors could contribute to recurrent and invasive breast cancer growth after lumpectomy, which suggests that the activity state of the two signaling pathways should be investigated in DCIS patients.

## Abbreviations

DCIS: ductal carcinoma *in situ*; DMEM: Dulbecco's modified Eagle's medium; EGF: epidermal growth factor; EGF: epidermal growth factor receptor; ERK: extracellular-signal regulated kinase; GFP: green fluorescence protein; 4-HT: 4-hydroxytamoxifen; MEK: mitogen-activated protein kinase kinase; PBS: phosphate-buffered saline; PI-3K: phosphoinositide-3 kinase.

## Competing interests

The authors declare that they have no competing interests.

## Authors' contributions

GWP conceived of the study, designed and performed the experiments, and wrote the manuscript. TH wrote the manuscript. Both authors read and approved the final manuscript.

## Acknowledgements

The authors thank Gina Yanochko and members of the Hunter laboratory for useful discussions. The vector pBABE-Raf:ER was a gift from Michael White and Ron Bumeister (University of Texas Southwestern Medical Center at Dallas, USA) and pCLNRX-H2B:GFP was a gift from Ee Tsin Wong and Geoff Wahl (Salk Institute). The work was supported by grants T32CA009370 and a Genentech Foundation Fellowship (to GWP), and CA14195 and CA82683 (to TH) from the National Cancer Institute. TH is a Frank and Else Schilling American Cancer Society Research Professor.

## Supplementary Material

Additional file 1 A Quicktime file containing a movie of a control acinus. The data file movie shows images taken at 30-minute intervals over 20 hours of imaging beginning 20 hours after the indicated treatment. The playback rate is 15 frames per second. The H2B:GFP-labeled nuclei are seen in white. The additional data file movies are the data used to generate the cell tracks shown in Figure 5b. Click here for file

Additional file 2 A Quicktime file containing a movie of an acinus treated with 100 nM 4-HT. The data file movie shows images taken at 30-minute intervals over 20 hours of imaging beginning 20 hours after the indicated treatment. The playback rate is 15 frames per second. The H2B:GFP-labeled nuclei are seen in white. The additional data file movies are the data used to generate the cell tracks shown in Figure 5b. Click here for file

Additional file 3 A Quicktime file containing a movie of an acinus treated with 100 nM 4-HT and 50 μM LY294002. The data file movie shows images taken at 30-minute intervals over 20 hours of imaging beginning 20 hours after the indicated treatment. The playback rate is 15 frames per second. The H2B:GFP-labeled nuclei are seen in white. The additional data file movies are the data used to generate the cell tracks shown in Figure 5b. Click here for file
